# The Role of Nonprofit–Private Collaboration for Nonprofits’ Organizational Resilience

**DOI:** 10.1007/s11266-021-00424-9

**Published:** 2021-10-26

**Authors:** Rebecca Waerder, Simon Thimmel, Benedikt Englert, Bernd Helmig

**Affiliations:** Chair of Business Administration, Public and Nonprofit Management, L 5, 4, University of Mannheim, 68161 Mannheim, Germany

**Keywords:** Resilience, Nonprofit–private collaboration, Refugees, Multiple case study, Resource dependence theory

## Abstract

Growing social, political, and economic uncertainties have shown that organizational resilience is becoming increasingly important for nonprofit organizations (NPOs). To ensure their long-term survival, NPOs need to respond to extreme events and adapt their services and processes. The theoretical premise of resource dependence theory assumes that interactions between an organization and its environment are crucial for the long-term adaptation to adversities. The present study investigates the contributions of nonprofit–private collaborations to organizational resilience of NPOs in light of the refugee crisis in Germany in 2015. Findings from a multiple holistic case study design indicate that collaborations of nonprofits with for-profit organizations support NPOs with stability, resources, expertise, and compassion to overcome resource-based, conceptual, and emotional challenges.

## Introduction

Climate action failure, unemployment, and large-scale involuntary migration are some of the most significant environmental, economic, and social challenges of the twenty-first century according to the Global Risk Report (World Economic Forum, 2020). Directing their services at socially marginalized groups or endangered environments, nonprofit organizations (NPOs) are strongly committed to overcoming these challenges. Thereby, they play an important role in mitigating these threats (McDougle & Lam, 2014). At the same time, social, political, and economic uncertainties lead to an unstable and rapidly changing environment hampering the work of NPOs (Witmer & Mellinger, 2016). Thus, NPOs need to continuously adapt to changing environments in order to continue their work in unstable settings (Mutongwizo, 2018). Organizations that successfully respond to adversities, effectively recover from extreme events, and expand their services in unstable environments are considered to be resilient and are therefore able to ensure their long-term survival under adverse conditions (Lengnick-Hall et al., 2011).

Organizational resilience of NPOs is often triggered by an extreme event that “[cannot] be addressed by routine measures” (Comfort & Kapucu, 2006, p. 310). One of the most recent extreme events challenging NPOs in Europe was the unanticipated arrival of a vast number of refugees in autumn 2015 (Simsa et al., 2019). Characterized as a “low-probability, high-impact event that threatens the viability of the organization” (Pearson & Clair, 1998, p. 60), the stream of refugees can be considered a crisis for NPOs. As a result, the economic, social, and political working environment for organizations changed rapidly (Simsa & Rothbauer, 2016). During this period, the work of NPOs was indispensable to facilitate the initial reception and accelerate the integration of thousands of refugees (Beck, 2016; Meyer & Simsa, 2018). Consequently, NPOs had to deal with an increased level of uncertainty and growing demand for their services at the same time. This challenge was even reinforced by information deficits, social polarization, and limited political intervention (Simsa & Rothbauer, 2016). However, a considerable number of NPOs were able to successfully respond to the disturbances of this extreme event, adapt to the unexpected situation, and sustain their mission during and in the aftermath of the refugee crisis.

As extreme events often demand “resources and skills from a wider range of organizations” (Comfort & Kapucu, 2006, p. 310), intersectoral collaborations become increasingly important for the recovery from extreme events and adaptation to unstable environments. In such times, NPOs need to deal with diminishing resources and a rapidly increasing demand for their services (Sanzo et al., 2015). In particular, intersectoral collaborations are important for NPOs’ resilience in the aftermath of the refugee crisis. By collaborating with for-profit organizations, NPOs can acquire resources, gain additional expertise, and develop new capabilities needed to cope with an elevated demand for their services (Schiller & Almog-Bar, 2013). Crucial enablers for the creation of collaborative alliances after extreme events are mutual goals and sharing of resources (Curnin & O'Hara, 2019).

So far, empirical studies on organizational resilience of NPOs revealed an increased competition for resources. Scholars stress the importance of relationships to other organizations for the acquisition of resources in rapidly changing environments (Gilstrap et al., 2016; Mutongwizo, 2018; Pape et al., 2019; Witmer & Mellinger, 2016). Additionally, research on interorganizational networks (Doerfel et al., 2013; Jung et al., 2019; Opdyke et al., 2017) and the establishment of intersectoral collaboration for disaster recovery (Comfort et al., 2001; Curnin & O'Hara, 2019; Simo & Bies, 2007) has examined to what extent organizational resilience is influenced through collaborations with other parties. However, these studies focus on the benefits of interorganizational collaborations in the aftermath of extreme events and do not reveal to what extent pre-existing collaborations affect the resilience of individual NPOs.

Recent studies on the role of NPOs during the refugee crisis in Europe have examined the relation between organizational characteristics and NPOs’ responses to the refugee crisis (Meyer & Simsa, 2018) or investigated the interplay between social volunteers and NPOs in overcoming the challenges of the refugee crisis (Fehsenfeld & Levinsen, 2019; Simsa et al., 2019). These studies have shown an increased demand for resources and the need for NPOs to adapt to the changing environments during the refugee crisis rapidly. However, there is yet no understanding of how resources and expertise from such nonprofit–private collaborations (NPCs) contribute to organizational resilience of NPOs in the aftermath of the refugee crisis. To address this research gap, we aim at answering the following research question: *How do NPCs Contribute to Organizational Resilience of NPOs?*

The contributions of this paper are threefold. First, we investigate the role of pre-existing NPCs for organizational resilience of NPOs, thereby countering the “abundance of valuable case studies” (van der Vegt et al., 2015, p. 974) and expanding the small number of empirical studies on organization resilience of NPOs (Mutongwizo, 2018; Witmer & Mellinger, 2016) by using a resource dependence perspective. In doing so, we complement studies that provide valuable insights into the establishment of intersectoral collaborations in the aftermath of extreme events (Doerfel et al., 2013; Jung et al., 2019; Opdyke et al., 2017). Second, our study adds to the literature on the reasons and benefits of NPCs in terms of resource and knowledge sharing (Arenas et al., 2013; Schiller & Almog-Bar, 2013; Sowa, 2009). Investigating the contributions of NPCs to organizational resilience offers a new perspective on the functioning of NPCs under adverse conditions and expands knowledge on the role of intersectoral collaborations for the long-term survival of NPOs. Third, by focusing on the role of NPCs for nonprofit resilience during the refugee crisis 2015, we additionally add to existing studies on NPO responses to the challenges of the stream of refugees in Europe and how NPOs successfully manage to cope with these (Fehsenfeld & Levinsen, 2019; Meyer & Simsa, 2018; Simsa et al., 2019).

## Theoretical Background

### Organizational Resilience of Nonprofit Organizations

Organizations that successfully adapt to extreme events and are able to adjust and maintain their functioning under adverse conditions are considered to be resilient (Williams et al., 2017). Even though literature provides numerous definitions of resilience, a general distinction can be made between the interpretation of resilience as a process or an outcome (Manyena, 2006). While the outcome perspective defines resilience as the ability to rebound from adversities and return to a status quo (Lengnick-Hall et al., 2011), the definition of resilience as a process exceeds the mere restoration of a status quo, indicating that organizations can create opportunities or develop new capabilities from extreme events to turn toward a more valuable state than before (Annarelli & Nonino, 2016; Williams et al., 2017). With regard to NPOs, organizational resilience is additionally determined by an organization’s commitment to its mission under adverse conditions (Witmer & Mellinger, 2016).

Extreme events “put extreme demands on the resources and process[es] of organizations” (James, 2011, p. 934). Hence, the development of organizational resilience often depends on an organization’s ability to obtain and retain resources (Vogus & Sutcliffe, 2007). To secure the acquisition of resources and thus ensure their long-term survival, “organizations are never self-sufficient but are interdependent with other organizations in their environment” (Helmig et al., 2014, p. 1515). These interdependencies of organizations form the basis of resource dependence theory (RDT), initially developed by Pfeffer and Salancik (1978). Focusing on the relation of an organization to its environment, RDT postulates that organizations interact with each other to acquire resources and thereby ensure their long-term survival (Pfeffer, 2005). The importance of interorganizational relations for the acquisition and exchange of resources becomes particularly evident in unstable and challenging settings that threaten the survival of organizations. This demands new strategies for dealing with limited resources from NPOs (Doyle et al., 2016; Malatesta & Smith, 2014). In this regard, alliances with private-sector firms have been identified as one of the strategies to pool resources, share information, and thereby enhance adaptability to adversities (Pape et al., 2019).

Focusing on the importance of resources and capabilities, empirical studies prove that organizational resilience exceeds the sum of individual resilience and includes inherent characteristics of organizations able to recover from extreme events and adapt to adversities (Linnenluecke, 2017). Hitherto, studies on organizational resilience focus on organizational reactions to changing funding or policy environments (Gilstrap et al., 2016; Mutongwizo, 2018; Pape et al., 2019). On the one hand, personnel resources, as well as skills, information, and expertise enable the adjustment to adversities and are therefore considered to enhance organizational resilience (Mutongwizo, 2018; Williams et al., 2017). On the other hand, studies from the for-profit sector indicate that an organization’s financial reserves facilitate the adaptation to adversities and enhance recovery from extreme events (Gittell et al., 2006). Moreover, existing research has acknowledged the importance of resource and information sharing for effective disaster recovery and adaptation to adversities (Boin et al., 2010; Comfort et al., 2001; Simo & Bies, 2007).

### Nonprofit–Private Collaboration

To obtain complementary resources crucial for their survival, NPOs engage in intrasectoral (Zeimers et al., 2019) as well as intersectoral collaborations (Atouba & Shumate, 2020; Chapman & Varda, 2017; Doyle et al., 2016). Particularly since companies have increasingly focused on CSR activities, collaborations with for-profit organizations have gained importance for NPOs to ensure the acquisition of resources, knowledge, and expertise (Schiller & Almog-Bar, 2013).

Nowadays, NPCs have gained considerable attention from various researchers (Seitanidi & Crane, 2009; Witesman & Heiss, 2017). As one form of intersectoral collaboration, they are generally defined as “the linking or sharing of information, resources, activities, and capabilities by organizations in two or more sectors to achieve jointly an outcome that could not be achieved by organizations in one sector separately” (Bryson et al., 2006, p. 44). NPCs can take various forms from charitable donations (“philanthropic stage”) and the exchange of resources (“transactional stage”) to an integrative form of collaboration where organizational objectives and processes merge into one integrated collective action (Austin, 2000).

The form of collaboration between NPOs and private-sector firms determines the benefits NPOs can obtain through NPCs (Wymer & Samu, 2003). While the acquisition of financial resources is considered the main reason for the establishment of NPCs (Schiller & Almog-Bar, 2013), strategic alliances, though being less common, go beyond the mere provision of financial resources and have the potential to increase social value (Sanzo et al., 2015). If NPCs follow strategic objectives toward a mutual social goal, the relationship between the partners exceeds a donor-recipient relationship and includes the exchange of more specialized resources that facilitate knowledge sharing. As nonprofit organizations are confronted with potentially different institutional logics in such more integrated collaborations, the processes and activities of both partners must be clearly aligned in order to avoid mission drift (Ebrahim et al., 2014). It is a matter of reconciling economic and financial value with social effectiveness (Bagnoli & Megali, 2011). This is not only important for achieving the social mission, but also strengthens the legitimacy within the context of cross-sector collaboration (Huybrechts & Nicholls, 2013). Such strategic collaborations eventually enable NPOs to deliver social value and accomplish their organizational goals (Sowa, 2009).

Existing studies on NPCs mainly address the benefits and forms of intersectoral collaborations under stable conditions. Yet, extreme events change the requirements of collaborations and shifts the mode and purpose of collaboration (Bryson et al., 2006). Since the maintenance of social objectives and the long-term survival of a NPO depend on its ability to acquire resources, the need to pool resources and share expertise becomes even more important in situations after extreme events that are characterized by uncertainty and resource scarcity (Comfort & Kapucu, 2006; Sowa, 2009).

With regard to the principles of collaborations under extreme events, researchers paid substantial attention to the role of intersectoral collaboration for disaster recovery (Boin et al., 2010; Comfort et al., 2001; Simo & Bies, 2007). On the one hand, the recovery from major extreme events often involves the participation of NPOs (Curnin & O'Hara, 2019). On the other hand, NPOs affected by extreme events are confronted with limited resources and a lack of information, thus suffering themselves from adversity (Comfort & Kapucu, 2006). To tackle these challenges and to recover from extreme events, NPOs collaborate with other organizations in their environment. With the following case study, we aim to understand better how exactly and under what context conditions NPCs contribute to the resilience of NPOs.

## Methods

### Design and Sampling Strategy

Due to the exploratory nature of our research question, we conducted a multiple holistic case study design (Yin, 2018). The chosen design enables an extensive investigation of the contemporary and complex phenomenon of organizational resilience and allows to detect commonalities and differences between the individual cases (Bryman & Bell, 2015). Thus, we included four NPOs as units of analysis. Considering the contextual dimension as an important feature of case studies, we investigated organizational resilience of NPOs in light of the refugee crisis in Germany in 2015.

**Fig. 1:**
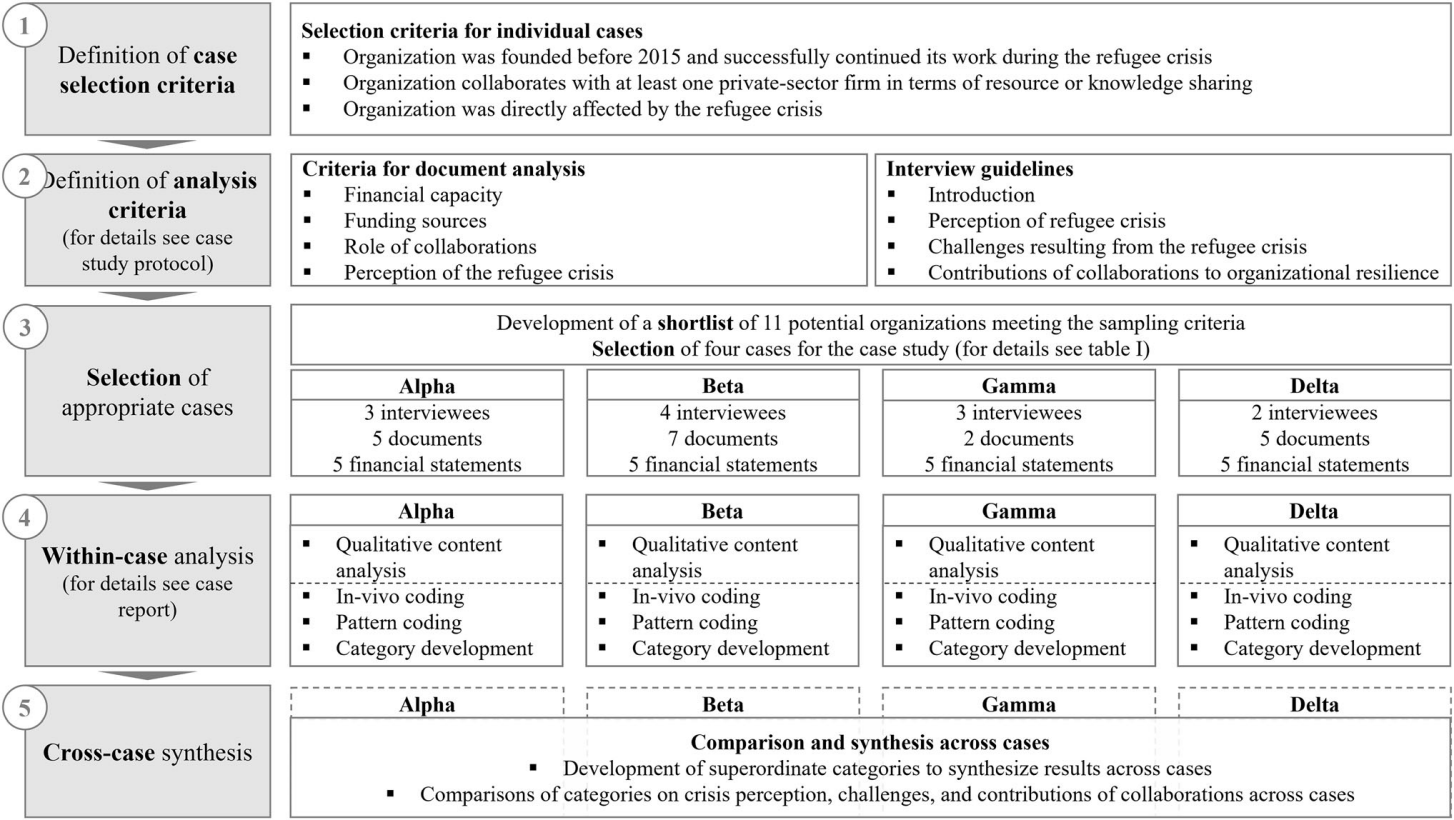
Research design

Figure 1 displays our research design, including the approach to data collection and analysis. To increase construct validity, we applied data triangulation (Yin, 2018). To this end, in-depth interviews within each organization were conducted and triangulated with data from organizational documents. The analysis of organizational documents focuses on the investigation of the context for each individual case. We increased the reliability of the case study by using a case study protocol (Yin, 2018). It contains detailed information about the data collection procedures, the evaluation criteria for organizational documents, and interview guidelines. The results of the analysis of organizational documents and interview data for each of the four cases individually can be retraced through the case report. Additionally, we stored interview transcripts and category systems for each organization in a case study database (CSDB). For data protection purposes, original organizational documents are not included in the CSDB and organization-specific information is anonymized in all documents. The case study protocol, case report, and CSDB are available on https://osf.io/upyqt/.

To select NPOs, we applied a purposive sampling strategy (Patton, 2009; Ritchie et al., 2014). To assure an in-depth investigation of the research question, NPOs were selected based on the following three criteria. First, the organizations were established before 2015 and successfully managed to continue their work and sustain their mission during and in the aftermath of the refugee crisis. Second, the organizations collaborated with at least one private-sector firm and established this alliance before 2015. Considering RDT, both transactional and integrative forms of collaborations between NPOs and private-sector firms were considered as they imply the exchange of knowledge and resources. Third, organizations were significantly affected by the stream of refugees in the form of increased demand or the need to adapt their service portfolios. An overview of the final sample is illustrated in Table 1.

**Table 1:** Final sample for the case study

Case	Region	Founded	Form of collaboration	Projects during the refugee crisis	Type of interview	Number of interviewees	Interview partner
Alpha	Rhine-Neckar	2008	Integrative	Job placements Mentoring programs Language classes	Individual interview	3	A1: Local manager A2: Manager A3: Project coordinator
Beta	Lower Rhine	1920	Transactional	Migrant counseling Refugee counseling Integration assistance	Group interview	4	B1: Employee migration service B2: Employee migration service B3: Employee migration service B4: Employee migration service
Gamma	Lower Rhine	1984	Integrative	Coaching for refugees Mentoring program Support center for refugees Initial reception of refugees	Individual interview	3	C1: Project coordinator C2: Local manager C3: Employee migration service
Delta	Rhine-Neckar	1953	Transactional	Medical aid for refugees Initial reception of refugees	Group interview	2	D1: Local manager D2: Volunteer

### Data Collection

Primary data were collected through open-ended semi-structured interviews (Yeo et al., 2014). To assure a holistic investigation of the research question, the twelve interview partners were selected based on their potential to contribute to our research objectives. Interviews were conducted case by case starting with Alpha in December 2019 and finishing with Delta beginning of February 2020. Based on the preferences of the interview partners, we conducted individual or group interviews. For group interviews, we applied scene setting and assured each participant was given sufficient time to answer each question in order to enable a balance of individual contributions (Finch et al., 2014).

The development of the interview guidelines was guided by the literature on organizational resilience and nonprofit private collaborations. We include the detailed interview guidelines in the case study protocol (https://osf.io/upyqt/). All interviews started with an introductory section on the participants position and task within the organization. In the main part, we focused on the organization’s view on the refugee crisis (Which challenges did you as an organization face during the refugee crisis in 2015?) and the role of collaborations (How did collaborations with private-sector firms actually look like during the refugee crisis?). To conclude interviews, we asked participants to describe how their organizations managed to meet the challenges resulting from the stream of refugees.

For triangulation purposes, annual reports covering the time span between 2014 and 2018, as well as additional organizational documents related to the refugee crisis or NPCs, were analyzed as secondary data (Yin, 2018). The interview data were interpreted against the background of the document analysis of each individual case. The use of different data sources allowed us to test the consistency of results (Patton, 2009) and to strengthen the case study by increasing construct validity (Bryman & Bell, 2015). The combination of in-depth interviews and organizational documents is also considered highly complementary (Yin, 2018) and therefore enabled us an information-rich, contextual investigation and in-depth understanding of the research subjects (Patton, 2009). In total, we collected 343 min of in-depth interviews and 671 pages of organizational documents, including annual reports and financial statements.

### Data Analysis

We applied cross-case synthesis for data analysis, as illustrated in Fig. 1. Thereby, we could gain a profound understanding of the individual cases but were also able to compare between the cases (Yin, 2018). In a first step, we examined the contributions of NPCs to organizational resilience for each individual case. Results of this within-case analysis are summarized in a case report and can be found on https://osf.io/upyqt/. Second, results were compared and analyzed across cases in order to synthesize individual case patterns and thus obtain a holistic understanding of the research topic (Yin, 2018).

**Fig. 2:**
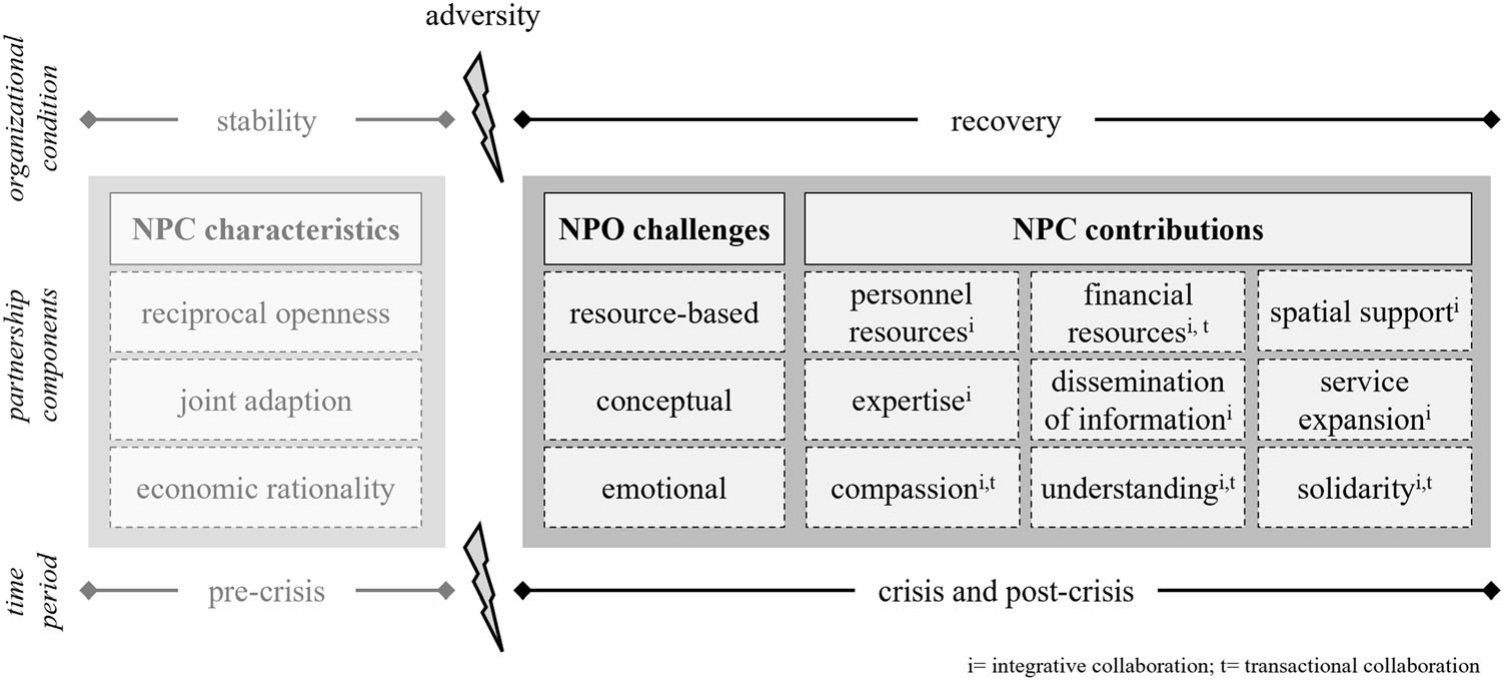
Cross-case synthesis

To analyze qualitative interviews, we adopted an inductive–deductive coding approach. As a first-cycle coding method, in vivo coding was applied to identify relevant statements directly from the participant’s language and accurately reflect the individual’s perception of and attitudes toward the research topic (Saldaña, 2013). To detect recurring statements, to classify categories, and to search for explanations within the data, pattern coding was deployed as a second-cycle coding method (Saldaña, 2013). Throughout this process, the coding scheme was continuously developed and further differentiated. Disagreements were continuously discussed until resolved (O’Connor & Joffe, 2020). In addition, throughout the analysis, we took the distinction between transactional and integrative forms of collaborations into account (see Fig. 2).

To examine the context for each individual case, we assessed organizational documents based on the following evaluation criteria: (1) financial capacity, (2) funding sources, (3) role of collaborations, and (4) perception of the refugee crisis. The investigation of the financial capacity is justified by the fact that the development of resilience demands additional financial resources from an organization (Bowman, 2011). Hence, financial capacity as the “resources that give an organization the wherewithal to seize opportunities and react to unexpected threats” (Bowman, 2011, p. 38) supports NPOs in becoming resilient and maintaining their mission in the long run. As privately funded NPOs are generally considered to be less vulnerable to extreme events (Hodge & Piccolo, 2005), the main sources of revenue for NPOs were additionally examined as a second indicator for financial stability. Moreover, we conducted a qualitative content analysis (Bryman & Bell, 2015) with regard to the perception of the refugee crisis and the role of collaborations with private-sector firms to subsequently triangulate results derived from the in-depth interviews. The perceived challenges of the crisis—conceptual, resource-based, and emotional ones—serve as a basis for examining the contributions of NPCs to organizational resilience. Thus, before detailing specific NPC contributions, we first introduce more general perceptions of the crisis in the results section.

## Results

### NPOs’ Crisis Perception and Challenges

Independent of their location or services, all cases perceived the stream of refugees as an extreme event. Even though NPOs were well aware of an increasing number of refugees, they did not expect the development “that fast and over that number” (local manager Alpha, 12/2019). Hence, the refugee crisis was mainly characterized by uncertainty and unexpectedness, thus requiring “immediate reaction over night” (employee migration service Gamma, 01/2020) and adaptation of NPOs. On the one hand, NPOs directly involved in emergency relief activities for refugees (Gamma, Delta) had to adapt to a new and unknown field of work. Those organizations were responsible for the medical treatment or initial reception of refugees at refugee shelters. On the other hand, NPOs focusing on legal and psychological consulting (Beta) or the integration of refugees into the labor market (Alpha, Gamma) aimed at sustainably integrating this additional target group into their services and therefore had to adjust existing programs to the specific needs of refugees. Yet, despite the perception of the refugee crisis as an extreme event, NPOs considered the situation a “new opportunity” (local manager Alpha, 12/2019) to establish collaborations, expand their target groups, and strengthen their position.

While responsibilities during the refugee crisis varied between the cases, all NPOs perceived the stream of refugees as a humanitarian crisis rather than a political or economic shock leading to similar challenges for NPOs. First, the major challenge refers to the increased demand for financial, personnel, and spatial resources required for the adjustment and implementation of programs with an increased number of participants. Second, conceptual challenges are directly related to the adaptation of programs and services, i.e., dealing with language barriers or adjusting programs to the specific needs of refugees. Lastly, emotional challenges reflect the burden for employees resulting from the work with psychologically stressed refugees. Moreover, this category includes internal as well as external skepticism NPOs faced when integrating refugees into their programs.

### Contributions of NPCs to NPOs’ Organizational Resilience

Figure 2 summarizes the contributions of NPCs to organizational resilience during the initial response to extreme events and the long-term adaptation and expansion of services across cases. Additionally, Table 2 provides empirical examples of the contributions of NPCs from the data. Although NPOs faced similar adversities and challenges, the contributions of NPCs to organizational resilience varied between the cases.

**Table 2:** Empirical examples of NPC contributions

NPO challenges	NPC contributions		
Resource-based	*Personnel resources*	*Financial resources*	*Spatial support*
	“We also have that with the mentoring, that some companies say that's working time, so they really credit the two hours a week that they spend with their participants” (manager Alpha 12/2020)	“Companies have then simply given a financial donation” (project coordinator Gamma 01/2020)	“We have a meeting room here, you can use it for your workshops” (manager Alpha 12/2020)
Conceptual	*Expertise*	*Dissemination of information*	*Service expansion*
	“We received a lot of feedback, so we adjusted and improved the program for young refugees. I think it was also very important to take along these players, because they all bring different positions, expertise and have also given us very, very valuable tips (local manager Alpha 12/2019)	“And there are companies that say: ‘The organization will come on Friday and will present in one hour what they do and what they are looking for. Interested people just come and see them.’ And then you have the managing director or head of personnel standing there, who says he supports the project and the company thinks the project is great. Then the employees have a completely different view on the issue. It has to be approved, so to speak” (local manager Alpha 12/2019)	“But there were also a lot of new companies that jumped in and said: ‘I'll do that and I'm willing to take a risk’” (local manager Alpha 12/2019)
Emotional	*Compassion*	*Understanding*	*Solidarity*
	“Companies came and asked if there was anything else they could do” (employee migration service Gamma 01/2020)	“So this is now a topic that not only concerns me as a nonprofit organization, but also companies” (project coordinator Alpha 12/2019)	“That you manage it hand in hand and that you are somehow in close consultation, in close cooperation, also in such a situation” (project coordinator Alpha 12/2019)

**Resource-based challenges:** Independent of their responsibilities during the refugee crisis, NPOs had to cope with resource scarcity, particularly affecting personnel resources, so that they “had to fall back on everyone available” (local manager Delta, 02/2020). On the one hand, NPOs managed an immense workload due to an increased demand for their services. On the other hand, organizations were confronted with a lack of qualified and trained personnel at the same time. NPOs maintaining integrative forms of collaboration (Alpha, Gamma) obtained additional **personnel resources** from private partners, not only including the access to the workforce but also the “provision of mentors or experts” (project coordinator Alpha, 12/2019) for the development and implementation of specific programs. In contrast, transactional collaborations only marginally supported NPOs (Beta, Delta) in countering resource scarcity as NPCs were not considered decisive for their work, thus being neglected due to the pressing challenges of the refugee crisis. For NPOs directly involved in emergency relief activities, collaborations were additionally hampered by legal and formal particularities of the crisis, i.e., changing legal frameworks or specific requirements for volunteers. Yet being present in all cases, **financial support** from NPCs was considered to be of minor importance since NPOs dealing with refugees could rely on increased private donations and obtained additional public grants or financial support from social lotteries. Still, the implementation of new programs for an additional target group demanded **spatial resources** from NPOs. In this regard, private partners “provided meeting rooms” (manager Alpha, 12/2019) or conducted projects at their company sites to support NPOs.

**Conceptual challenges:** As new programs for refugees had to be “built up completely from scratch” (project coordinator Gamma, 01/2020), NPOs often demanded additional skills and input. In this regard, for-profit partners supported NPOs with their professional **expertise** for the development of specific programs, such as industry-specific language courses. Furthermore, the joint development of programs implied a continuous exchange between the two parties, thus enabling NPOs to gain feedback or an external perspective on the development of their programs. In contrast to the other cases, Alpha indicates the importance of NPCs for the **dissemination of information**. In order to support the NPO, companies internally distributed information to potential mentors or experts and externally served as a reference example for the acquisition of new partners. Lastly, private partners viewed the refugee crisis from an economic perspective and recognized the advantages of gaining a skilled workforce to counter skills shortages. Thereby, companies contrasted the humanitarian perspective on the crisis and facilitated the integration of refugees into the labor market (Alpha). Thus, NPCs also supported organizations in adapting to the specific requirements of the refugee crisis and the stream of incoming people. Companies were open toward an unknown target group as well as new programs, thus supporting NPOs in the **expansion of their services**. Hence, NPOs and private partners joined forces and adapted to the refugee crisis “hand in hand” (local manager Alpha 12/2019).

**Emotional challenges:** The precarious and uncertain humanitarian situation of refugees led to an emotional burden for employees. They “worked far beyond personal limits” (employee migration service Beta 12/2019) with “considerable cuts in the private sphere of life” (local manager Gamma 01/2020), and struggled to cope with the “pictures of freezing people, that stay forever in mind” (local Manager Delta 02/2020). In this unstable environment, pre-existing NPCs form “a trusting collaboration” (employee migration service Gamma 01/2020) built on mutual appreciation. Consequently, NPOs benefited emotionally in terms of experienced **compassion** by their for-profit partners. Moreover, private partners showed their **understanding** of the exceptional and demanding situation of NPOs and were open to new programs as well as to new target groups. The NPOs witnessed great **solidarity** in form of joint adaption as the refugee crisis was a collective challenge that “not only concerns me as a NPO, but companies as well” (project coordinator Alpha 12/2019).

In summary, NPCs generally provided NPOs with stability and continuity during the uncertainties of the crisis, thereby facilitating the adaptation to adversities. Yet, the contributions of NPCs to organizational resilience varied between the different cases. NPOs in **integrative** NPCs aiming at the integration of refugees into the labor market considered the collaboration as decisive for the adaptation of their programs and particularly benefitted from the provision of mentors, the professional input, and the openness of companies. NPOs focusing on **transactional** collaborations rather obtained general support from NPCs, such as stability and continuity during the uncertainties of the refugee crisis. Regardless of the differences in contributions of NPCs to organizational resilience between the cases, NPCs were generally considered desirable and are gaining importance after the response to the initial shock of the refugee crisis.

## Discussion

Confronted with the adversities of the refugee crisis, NPOs need to adapt to changing working environments and additional requirements for their services (Meyer & Simsa, 2018). Hence, the transition from managing the initial shock of the refugee crisis to the long-term adaptation to uncertainties confirms that crisis management and organizational resilience are indispensably linked (Williams et al., 2017). The challenges resulting from the stream of refugees additionally show the ambiguous role of NPOs in the aftermath of extreme events. They contribute significantly to a societal response to the crisis (Curnin & O'Hara, 2019) while simultaneously dealing with obstacles of the crisis, such as uncertainty and resource scarcity (Comfort & Kapucu, 2006). Subsequently, we pick up the discussion of our findings about NPCs’ contributions to NPO resilience under consideration of the relevant context conditions shaping the contributions’ mode of action.

Contributions of NPCs to organizational resilience prove to be dependent on the involvement of NPOs in the public response to the refugee crisis. Initially, NPOs are often overwhelmed by an immense workload due to the reception of refugees, further measures for their long-term integration, and the coordination of volunteers (Simsa & Rothbauer, 2016). Hence, NPOs need to focus their resources on emergency relief activities, thereby exacerbating the maintenance of NPCs. In order to mitigate the immediate social consequences of the humanitarian crisis, these organizations establish collaborations with public partners (Meyer & Simsa, 2018), thus following their “orientation toward the public good” (Simo & Bies, 2007, p. 125). In contrast, NPCs rather gain importance with respect to the long-term adaptation of services and programs.

Moreover, the contributions of NPCs to organizational resilience vary with the form of collaboration between the partners. On the one hand, NPOs consider NPCs an integral part of their mission fulfillment and also perceive collaborations as a crucial element for the development of organizational resilience. During the refugee crisis, the role of the for-profit partner remains decisive as NPCs support NPOs in mitigating the consequences of the extreme event and adapting to adversities. In contrast, NPOs maintaining transactional collaborations rather focus on the exchange of knowledge and resources. Similar to the benefits NPOs can obtain from partnerships under “normal” conditions (Wymer & Samu, 2003), the contributions of transactional NPCs to organizational resilience therefore prove to be less substantial.

Although the degree of contributions of NPCs differs between cases, our findings generally reinforce the assumption that organizational resilience of NPOs depends on the availability of personnel and financial resources (Mutongwizo, 2018). In line with RDT, organizations are dependent on their environment in order to obtain resources (Pfeffer & Salancik, 1978). While social or economic crises are often characterized by resource scarcity, collaborations are considered decisive for the acquisition of resources during and in the aftermath of extreme events.

Similar to existing empirical studies (Opdyke et al., 2017), our research reveals that personnel resources can be considered the scarcest resource due to the rapidly growing demand and the lack of qualified human resources during the refugee crisis. On the one hand, the provision of personnel resources by private partners significantly contributes to organizational resilience of NPOs as it enhances adaptability (Vogus & Sutcliffe, 2007). On the other hand, extreme events demand effective coordination of personnel resources as employees and volunteers often require specific training (Comfort & Kapucu, 2006; Opdyke et al., 2017). Thus, private partners are not always able to provide NPOs with the resources needed for the adaptation to extreme events. Even though access to financial resources is deemed to be essential for the development of organizational resilience (Bowman, 2011; Mutongwizo, 2018), the acquisition of financial resources through NPCs is of minor importance for NPOs meeting the challenges of the refugee crisis since in such emergency situations the social service is at the core of action. Here, pre-existing NPCs that have developed social capital over time (Richards & Reed, 2015) can serve as an additional strength donor for NPOs through sole compassion, understanding, and solidarity.

The adaptation to adversities and expansion of services does not only demand additional resources but also requires specific capabilities (Williams et al., 2017). During the refugee crisis, NPOs were able to obtain feedback and expertise for the adjustment of their programs from NPCs. In addition, pre-existing collaborations are deemed to facilitate the exchange of information after extreme events (Simo & Bies, 2007) as political and legal frameworks constantly changed during the refugee crisis (Simsa & Rothbauer, 2016). In summary, we find that NPOs are able to acquire both additional resources and capabilities throughout crises (Vogus & Sutcliffe, 2007) that supported organizational resilience.

Our results also confirm that pre-existing collaborations endure extreme events and are even reinforced in the aftermath of the crisis (Curnin & O'Hara, 2019). Private partners’ openness in our cases toward new projects reflects the required flexibility of interorganizational collaborations in the aftermath of extreme events (Doerfel et al., 2013). Moreover, while diverging interests between NPOs and private-sector firms often harbor an increased potential for conflicts (Sanzo et al., 2015), the contrast between the humanitarian and economic perspective on the refugee crisis supported NPOs in sustainably integrating refugees. Hence, diverging perspectives facilitate the long-term expansion of their services.

Lastly, as a humanitarian crisis, the refugee crisis poses particular stresses and requirements on both individual partners and the collaboration. On an individual level, based on the generally precarious situation and individual fates of refugees, the crisis leads to an increased personal psychological and physical burden for volunteers and employees (Simsa & Rothbauer, 2016). Hence, these groups are at risk of experiencing secondary trauma symptoms due to their work (Elwood et al., 2011). Moreover, organizationally NPOs are confronted with legal particularities concerning the work with refugees. These official regulations often impede collaborations or pose additional challenges to NPOs and companies.

## Limitations and Future Research

As with all qualitative approaches, our study is not without limitations. First, we restricted our sample to resilience throughout the refugee crisis. However, rather than representational, we aimed at inferential generalizability (Lewis et al., 2014). Hence, based on our findings, we infer that identified contributions also play a major role in other crises. Yet, we believe that specific aspects might differ in significance and degree. For example, the Covid-19 coronavirus crisis puts higher financial pressures on NPOs creating a higher need for financial stability and support within NPC settings. Thus, we call for a replication of our design in other contexts than humanitarian crises (Helmig et al., 2012).

Second, also with regard to the generalizability of our findings, we only looked at successful collaborations. According to the core concept of our study, resilience, we can only discuss scientific findings about success factors in collaborations that helped nonprofit organizations to survive upcoming challenges. To counter and complement such survivorship bias, future studies should examine, how and why, despite being engaged in collaborations, some nonprofit organizations went under throughout the refugee crisis. This could be done via qualitative retrospective narrative approaches with the responsible parties involved (Ritchie et al., 2014).

Third, data were collected cross-sectionally, making it difficult to draw conclusions about long-term resilience and adaptation to future adversities. The exploratory design of the case study opens up opportunities for longitudinal studies on the contributions of NPCs to organizational resilience of NPOs as flight and migration remain challenging areas of action (Simsa & Rothbauer, 2016). Apparently, this also holds for future investigations on an individual level, questioning in how far the identified emotional challenges manifest over time. Longitudinally investigating our research context could help to sharpen the interpretation of resilience not only as recovery from extreme events but also as a long-term adaptation process to adverse environments (van der Vegt et al., 2015). Thereby, we could learn more about the concept’s dynamic nature.

Fourth, we only looked at one specific type of partnerships, namely NPCs. However, crises like the refugee crisis as a primarily humanitarian crisis also involve public actors. Besides looking into other contexts, future research might assess how NPOs collaborate with public partners to manage resource dependencies (Doyle et al., 2016). Such studies could assess how nonprofit-public partnerships (mutually) foster organizational resilience. Moreover, such research endeavors would allow for comparisons between the role of collaborations with public and private partners (Austin, 2000).

## Management Implications

As NPOs are particularly confronted with scarce personnel resources in the aftermath of extreme events, NPO managers should recognize the importance of NPCs for the recovery from adversities in order to reduce resource uncertainties and facilitate adaptation (Malatesta & Smith, 2014). Considering the stream of refugees as a humanitarian crisis, the exchange of resources became particularly evident in the provision of personnel resources and expertise. However, the acquisition of resources through NPCs is not limited to personnel resources but aims at supporting NPOs facing economic, social, or political burdens.

Moreover, NPOs are encouraged to focus on integrative forms of NPCs in order to assure the joint work of both partners toward a mutual goal (Austin, 2000). As the case study shows, long-lasting integrative relationships between NPOs and private partners are built on trust and mutual appreciation. Since both partners are committed to a joint mission and work toward a mutual goal, their relationship can even be strengthened by an extreme event. Yet, even if NPO managers hesitate and fear power imbalance in such a partnership: also transactional collaborations proved to be relevant as they enable the exchange of resources and can therefore facilitate adaptation to adversities (Austin, 2000). As the present study shows, the long-term goal and strategic importance of NPCs for an organization´s mission fulfillment was often neglected due to the pressing challenges of the situation. To prevent collaborations from shifting into a donor-recipient relationship and to ensure the accomplishment of long-term goals, NPO managers should explicitly negotiate and formally document the strategic objectives of NPCs. At the same time, nonprofit organizations should consider both the financial and social bottom line to ensure both economic stability and social mission fulfilment (Grieco et al., 2015; Mook et al., 2015). Cooperation can only be continuously developed through good documentation of the shared social impact.

Based thereupon, strategic collaborations can enhance adaptation and have the potential to sustainably support NPOs beyond the recovery from extreme events even though extreme events often require the immediate response and reaction to accompanied adversities.
